# Crystal structure of *meso*-di-μ-chlorido-bis­[bis­(2,2′-bi­pyridine)­cadmium] bis­(1,1,3,3-tetra­cyano-2-ethoxy­propenide) 0.81-hydrate

**DOI:** 10.1107/S205698901601971X

**Published:** 2017-01-01

**Authors:** Fatima Setifi, Bernd Morgenstern, Kaspar Hegetschweiler, Zouaoui Setifi, Rachid Touzani, Christopher Glidewell

**Affiliations:** a Laboratoire de Chimie, Ingénierie Moléculaire et Nanostructures (LCIMN), Université Ferhat Abbas Sétif 1, Sétif 19000, Algeria; b Fachrichtung Chemie, Universität des Saarlandes, Postfach 151150, D-66041 Saarbrücken, Germany; c Laboratoire de Chimie Appliquée et Environnement, LCAE-URAC18, COSTE, Faculté des Sciences, Université Mohamed Premier, BP 524, 60000, Oujda, Morocco; d Faculté Pluridisciplinaire Nador BP 300, Selouane, 62702, Nador, Morocco; e School of Chemistry, University of St Andrews, St Andrews, Fife KY16 9ST, UK

**Keywords:** crystal structure, hydro­thermal synthesis, polynitrile anions, mol­ecular structure, mol­ecular disorder, hydrogen bonding

## Abstract

The hydrated title salt, [Cd_2_Cl_2_(C_10_H_8_N_2_)_4_](C_9_H_5_N_4_O)_2_·0.81H_2_O, was obtained from the hydro­thermal reaction between 2,2′-bi­pyridine, cadmium(II) chloride and potassium 1,1,3,3-tetra­cyano-2-eth­oxy­propenide. The binuclear cation lies across a centre of inversion in the space group *P*2_1_/*c*, with the other components in general positions. The cation has approximate, but non-crystallographic 2/*m* symmetry and each of the Cd^II^ atoms is a stereogenic centre, one having the Δ configuration and the other the Λ configuration. In the anion, one of the C(CN)_2_ units is disordered over two sets of atomic sites having occupancies 0.75 (2) and 0.25 (2). The cations are linked by two independent C—H⋯Cl hydrogen bonds to form a sheet of *R*_2_^2^(14) and *R*_4_^2^(24) rings.

## Abstract 2

In the title compound, which was prepared using a hydro­thermal reaction between 2,2′-bi­pyridine, cadmium(II) chloride and potassium 1,1,3,3-tetra­cyano-2-eth­oxy­propenide, the complex cations are linked into sheets by C—H⋯Cl hydrogen bonds.

## Chemical context   

Luminescent materials based on transition metals and lanthanoids have found wide applications in lighting (Pust *et al.*, 2014 ▸), luminescence sensing (Liu *et al.*, 2015 ▸) and optical devices (Torres *et al.*, 2015 ▸). Among them, *d* ^10^ metal complexes comprising zinc(II) and cadmium(II) with a variety of ligands have attracted considerable attention in recent years because of their luminescence properties (Mautner *et al.*, 2015 ▸).

Organic polynitrile ligands are versatile structural components, leading to many different architectures in zero, one, two or three dimensions, and incorporating most of the 3*d* trans­ition metals (Miyazaki *et al.*, 2003 ▸; Yuste *et al.*, 2009 ▸; Benmansour *et al.*, 2010 ▸; Gaamoune *et al.*, 2010 ▸; Setifi *et al.*, 2013 ▸; Setifi, Setifi *et al.*, 2014 ▸; Addala *et al.*, 2015 ▸). The versatility of such ligands is based on two main properties: firstly, the ability to act as bridges, given the linear and rigid geometry of the cyano groups, and secondly, the possibility of combining these ligands with a wide variety of co-ligands, leading to an extensive variety of coordination modes. To take advantage of this behaviour, we have been using polynitrile anions in combination with other chelating or bridging neutral co-ligands to explore the structural and electronic characteristics of the resulting complexes, particularly with reference to mol­ecular materials exhibiting inter­esting luminescent behaviour.

Here we report the synthesis and structure of the title compound (I), the first dinuclear cadmium(II) coordination compound containing the organic polynitrile 1,1,3,3-tetra­cyano-2-eth­oxy­propenide counter-anion (abbreviated as tcnoet^−^) in combination with the chelating ligand 2,2′-bi­pyridine.
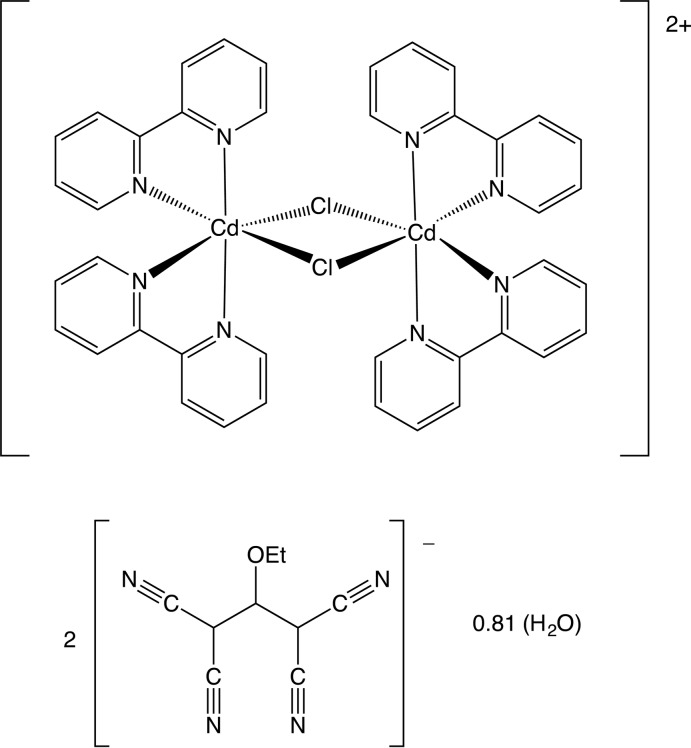


## Structural commentary   

**Figure 1:**
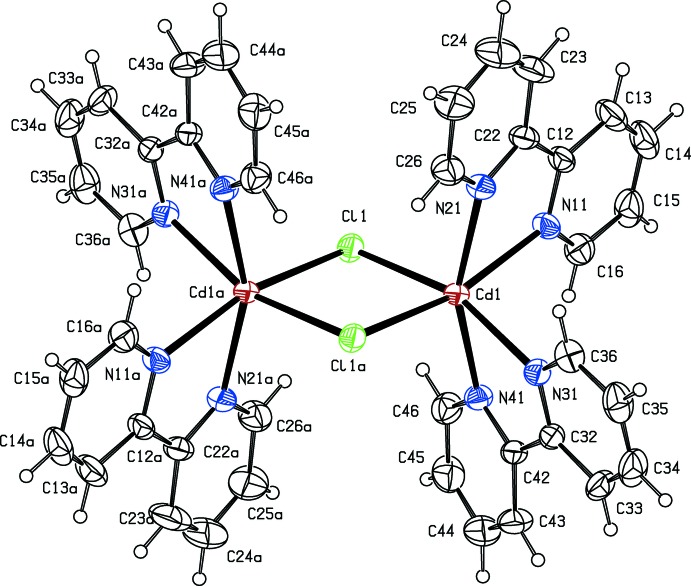
The structure of the binuclear cation in compound (I), with displacement ellipsoids drawn at the 30% probability level. Atoms marked with ‘a’ are at the symmetry position (−*x* + 1, −*y* + 1, −*z* + 1). Selected bond lengths (Å): Cd1—N11 2.358 (2), Cd1—N21 2.342 (2), Cd1—N31 2.341 (2), Cd1—N41 2.350 (2), Cd1—Cl1 2.5920 (9), Cd1—Cl1^a^ 2.6289 (8). Selected bond angles (°): N11—Cd1—N21 70.30 (8), N31—Cd1—N41 70.412 (8), Cl1—Cd1—Cl1^a^ 84.51 (3), Cd1—Cl1—Cd1^a^ 95.49 (3), N11—Cd1—Cl1^a^ 165.01 (6), N21—Cd1—N41 158.62 (8), N31—Cd1—Cl1 161.37 (6).

**Figure 2:**
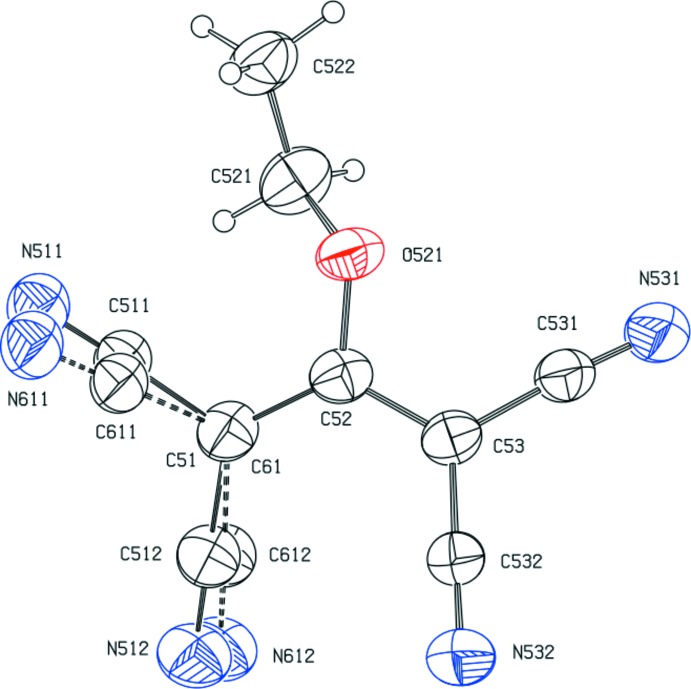
The structure of the anion in compound (I), with displacement ellipsoids drawn at the 30% probability level. Atomic sites C51 and C61 were constrained to be identical and the major and minor components of the disordered C(CN)_2_) unit are drawn with full and dashed lines, respectively.

The structure consists of a di-*μ*_2_-chlorido-bis­[bis­(2,2′-bi­pyridine)­cadmium] dication, [Cd_2_Cl_2_(C_10_H_8_N_2_)_4_]^2+^, which lies across a centre of inversion in space group *P*2_1_/*c* (Fig. 1 ▸), and a tcnoet^−^ anion, (NC)_2_CC(OEt)C(CN)_2_, which lies in a general position (Fig. 2 ▸). The reference cation was selected as that lying across (1/2, 1/2, 1/2). The structure also contains a partial occupancy water mol­ecule lying in a general position with refined occupancy 0.403 (6), but the partial occupancy H atoms associated with this could not be reliably located.

Within the cation, the Cd^II^ atoms are six-coordinate with the two bridging chlorido ligands occupying mutually *cis* sites. The *cis*-bidentate coordination geometry at Cd means that this atom is a stereogenic centre and the reference Cd atom was selected as the one having the Δ configuration. The inversion-related Cd atom within the binuclear cation thus has the Λ configuration, so that the cation represents a *meso* form. Overall, the cation has approximate, but non-crystallographic 2/*m* (*C*_2*h*_) symmetry, with the twofold rotation axis along the Cd⋯Cd vector and the mirror normal to this and containing the two chlorido ligands.

The Cd—N distances for the bonds *trans* to the bridging chlorido ligands do not differ markedly from the two Cd—N distances which are mutually *trans* (see Fig. 1 ▸). The two Cd—Cl distances are, however, significantly different. The inversion symmetry of the cation means that the central Cd_2_Cl_2_ ring is strictly planar, although it is not rectangular [Cl1—Cd1—Cl1^a^ = 84.51 (3)°; symmetry code: (a) −*x* + 1, −*y* + 1, −*z* + 1].

The six-coordinate geometry at the Cd atom is markedly distorted from an idealized octa­hedral geometry (Fig. 1 ▸), and the bond angles at Cd are probably dominated by the bite angles of the bipy ligands and the central ring geometry. Thus, because of the small bite of the 2,2′-bipy ligand, the N—N distances within these ligands, 2.705 (3) Å and 2.706 (4) Å, are significantly shorter than those along the remaining edges of the CdCl_2_N_4_ octa­hedron, which range from 3.387 (3) to 3.760 (3) Å; as a consequence, the torsional angle N11—Cl1—Cl1^a^—N31 is 21.3 (4)°, rather than the zero degrees expected for a regular octa­hedron. The structural motif of such a *meso*-[(CdClN_4_)_2_]^2+^ entity has been found in a variety of complexes with 1,2-di­amino­ethane (Näther & Jess, 2010 ▸), 1,10-phenanthroline (Wang *et al.*, 2012 ▸) and 3,5-di­methyl­pyrazole-1-carboxamidine (Holló *et al.*, 2009 ▸) as chelating ligands. The largest N—N separations [2.899 (5) Å and 2.909 (6) Å], are observed for the flexible ligand 1,2-di­amino­ethane, while for 1,10-phen and 3,5-di­methyl­pyrazole-1-carboxamidine the corresponding N—N separation is slightly smaller than in compound (I). In agreement with this observation, the N—Cl—Cl—N dihedral angle increases in the order: monodentate N-donors (Hu *et al.*, 2016 ▸) 3.5 (6)° < 1,2-di­amino­ethane 10.6 (4)° < 2,2′-bipy 21.3 (4)° < 1,10-phen 26.8 (7)° and 3,5-di­methyl­pyrazole-1-carboxamidine 26.4 (5)°.

One of the C(CN)_2_ groups in the tcnoet^−^ anion is disordered over two sets of atomic sites, with occupancies 0.75 (2) and 0.25 (2), which are related by a mutual rotation about the C—C bond to atom C52 (Fig. 2 ▸). The dihedral angles between the central plane (C51,C52,C53) and the major and minor components of the disordered C(CN)_2_ unit are 20.3 (6) and 31.6 (15)°, respectively, while the dihedral angle between the central plane and the ordered C(CN)_2_ unit is 17.1 (6)°, such that the rotations of two C(CN)_2_ units out of the central plane are in a conrotatory sense. The dihedral angle between the planes of the major and minor disorder forms is 12.4 (17)°. The C—N distances in the anion are all very similar, as are the corresponding values for the two types of C—C distances in the tetra­cyano­propenide portion, with their magnitudes pointing to extensive delocalization of the negative charge not only over the propenide unit but also into the cyano groups, as previously discussed (Setifi *et al.*, 2016 ▸).

## Supra­molecular inter­actions   

The supra­molecular assembly is determined by two independent C—H⋯Cl hydrogen bonds (Table 1 ▸). Database analyses (Brammer *et al.*, 2001 ▸; Thallypally & Nangia, 2001 ▸) have demonstrated that chlorido ligands bonded to metals are effective hydrogen-bond acceptors, even from weak donors such as C—H, and the two hydrogen bonds here link the reference cation centred at (1/2, 1/2, 1/2) to the four symmetry-related cations centred at (1/2, 0, 0), (1/2, 1, 0), (1/2, 0, 1) and (1/2, 1, 1), so generating a sheet lying parallel to (100) and containing hydrogen-bonded rings of 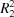
(14) and 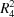
(24) types. The formation of the sheet is reinforced by a π–π stacking inter­action. The pyridyl ring containing atom N11, which lies in the cation centred at (1/2, 1/2, 1/2), makes a dihedral angle of only 1.78 (14)° with the pyridyl ring containing N31 at (*x*, 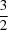 − *y*, 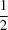 + *z*), which lies in the cation centred at (1/2, 1, 1). The ring-centroid separation is 3.602 (2) Å and the shorted perpendicular from the centroid of one ring to the plane of the other is 3.3878 (11) Å, corresponding to a ring-centroid offset of *ca* 1.22 Å.

The anions are linked to this sheet by C—H⋯N hydrogen bonds, but otherwise play no part in the supra­molecular assembly.

**Figure 3:**
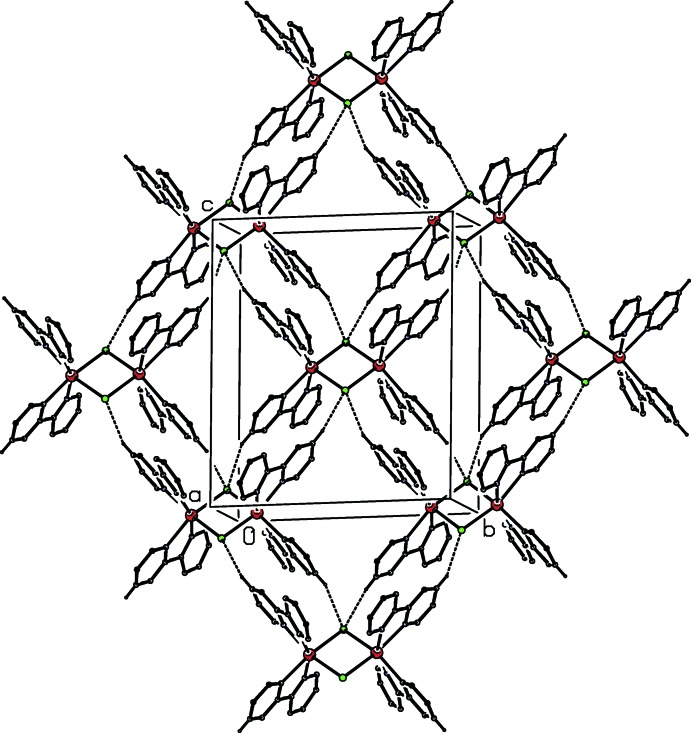
Part of the crystal structure of compound (I), showing the formation of a sheet of cations parallel to (100) built from two C—H⋯Cl hydrogen bonds and containing 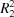
(14) and 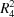
(24) ring motifs. For the sake of clarity, the anions and water mol­ecules, and those H atoms of the cation which are not involved in the motifs shown have been omitted.

The partial-occupancy atom O71 is linked to the cation by a C—H⋯O hydrogen bond (Table 1 ▸). Although the H atoms associated with atom O71 could not be located, nonetheless atom O71 is within plausible hydrogen-bonding distance of the N atoms, N511 and N611 both at (−*x*, −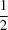 + *y*, 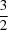 − *z*) and N532 at (*x*, −1 + *y*, *z*), with O⋯N distances 2.935 (4), 2.72 (4) and 3.186 (8) Å, respectively. The corresponding N⋯O⋯N angles involving the N atoms in the major and minor components of the disordered anion are 95.4 (3) and 105.5 (6)°, respectively. If these contacts represent hydrogen bonds, then that involving atom N532 lies within the sheet already described (Fig. 3 ▸), while the other two would combine to link these sheets into a three-dimensional framework structure.

## Database survey   

The structure of the tcnoet^−^ unit has been reported in salt-like compounds, both with organic cations (Setifi, Lehchili *et al.*, 2014 ▸; Setifi *et al.*, 2016 ▸) and with cationic metal coordination complexes (Gaamoune *et al.*, 2010 ▸; Setifi *et al.*, 2013 ▸), and as a coordinating ligand. Examples have been reported recently in which the tcnoet^−^ unit acts as both a bridging and a terminal ligand with Cu^II^, leading to the formation of a coordination polymer in the form of a ribbon (Addala *et al.*, 2015 ▸), and where it acts as a *μ*_3_-bridging ligand, also with Cu^II^, leading to the formation of a coordination polymer sheet (Setifi, Setifi *et al.*, 2014 ▸).

The structure of the dicadmium cation present in compound (I) appears not to have been reported previously. However, in the analogous cation [(μ_2_-Cl)_2_(en_2_Cd)_2_]^2+^, characterized as its chloride salt (Näther & Jess, 2010 ▸), the cation again lies across a centre of inversion, here in space group *P*2_1_/*n*, with a geometry at Cd very similar to that in compound (I). The related cation [(*μ*_2_-Cl)_2_(phen_2_Cd)_2_]^2+^ has been characterized in two polytungstate salts, one of them as a 4,4′-bi­pyridine solvate. In the unsolvated salt, the cation lies across a twofold rotation axis in *C*2/*c* (Wang *et al.*, 2011 ▸); by contrast, in the solvated salt (Wang *et al.*, 2012 ▸), the cation is almost centrosymmetric, although examination of the atomic coordinates using *PLATON* (Spek, 2009 ▸) suggests that the space group may be *P* 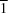 rather than the reported *P*1 (*cf.* Marsh, 1999 ▸, 2005 ▸, 2009 ▸). Finally, we note some neutral dicadmium complexes of type (*μ*_2_-Cl)_2_(ClCd*L*)_2_, where *L* represents a tridentate aliphatic amine ligand, which have mol­ecular architectures similar to that in the cation of compound (I): when *L* represents 2-amino­ethyl-3-amino­propyl amine (Gannas *et al.*, 1980 ▸) or *cis*-3,5-di­amino­piperidine (Pauly *et al.*, 2000 ▸), the complexes lie across inversion centres in space group types *P*2_1_/*n* and *P*2_1_/*c*, respectively, but when *L* represents bis­(3-amino­prop­yl)amine (Gannas *et al.*, 1980 ▸), the complex lies across a twofold rotation axis in *C*2/*c*.

## Synthesis and crystallization   

The salt K(tcnoet) was prepared using the published method (Middleton *et al.*, 1958 ▸). The title compound was synthesized hydro­thermally under autogenous pressure from a mixture of cadmium(II) chloride (40 mg, 0.21 mmol), 2,2′-bi­pyridine (32 mg, 0.21 mmol) and K(tcnoet) (90 mg, 0.40 mmol) in water–methanol (4:1 *v*/*v*, 20 cm^3^). This mixture was sealed in a Teflon-lined autoclave and held at 423 K for 2 d, and then cooled to ambient temperature at a rate of 10 K h^−1^ (yield 47%). Colourless prisms of the title compound suitable for single-crystal X-ray diffraction were selected directly from the synthesized product.

## Refinement   

Crystal data, data collection and structure refinement details are summarized in Table 2 ▸. Three low-angle reflections, (100), (011) and (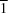
02), which had been attenuated by the beam stop, were omitted from the refinement. The H atoms bonded to C atoms were located in difference maps and then treated as riding atoms in geometrically idealized positions with C—H distances of 0.93 Å (pyridine), 0.96 Å (CH_3_) or 0.97 Å (CH_2_) and with *U*_iso_(H) = *kU*_eq_(C) where *k* = 1.5 for the methyl group, which was permitted to rotate but not to tilt, and 1.2 for all other H atoms bonded to C atoms. It was apparent from an early stage that the cyano groups in one of the C(CN)_2_ units of the anion, that containing atom C51, are disordered over two sets of atomic sites having unequal occupancies. For the minor disorder form, the bond lengths and the 1,3 non-bonding contacts were restrained to be the same as the corresponding distances in the major form, subject to s.u. values of 0.005 and 0.01 Å, respectively. In addition, the anisotropic displacement parameters for pairs of partial-occupancy atoms occupying essentially the same physical space were constrained to be identical. Subject to these conditions, the occupancies of the major and minor disorder forms refined to 0.75 (2) and 0.25 (2). For the partial-occupancy water mol­ecule, the atomic coordinates of the O atom were refined with *U*_iso_(O) fixed at 0.08 Å^2^, giving a refined occupancy of 0.403 (6). A difference map provided plausible locations for two H atoms associated with this O atom but neither of these sites was within hydrogen-bonding range of any likely acceptor and hence they were probably just artefacts of the isotropic refinement. In the final analysis of variance, there was a negative value, −0.835, of *K* = mean(*F*_o_^2^)/mean(*F*_c_^2^) for the group of 1177 very weak reflections having *F*_c_/*F*_c_(max) in the range 0.000 < *F*_c_/*F*_c_(max) < 0.006.

## Supplementary Material

Crystal structure: contains datablock(s) global, I. DOI: 10.1107/S205698901601971X/wm5346sup1.cif

Structure factors: contains datablock(s) I. DOI: 10.1107/S205698901601971X/wm5346Isup2.hkl

CCDC reference: 1521824

Additional supporting information: crystallographic information; 3D view; checkCIF report

## Figures and Tables

**Table 1:** Hydrogen-bond geometry (Å, °)

*D*—H⋯*A*	*D*—H	H⋯*A*	*D*⋯*A*	*D*—H⋯*A*
C14—H14⋯Cl1^i^	0.93	2.79	3.651 (4)	154
C15—H15⋯N512	0.93	2.63	3.456 (17)	149
C15—H15⋯N612	0.93	2.46	3.29 (5)	149
C34—H34⋯Cl1^ii^	0.93	2.82	3.705 (4)	160
C46—H46⋯O71	0.93	2.49	3.292 (8)	145

Symmetry codes: (i) 
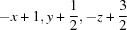
; (ii) 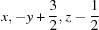.

**Table 2:** Experimental details

Crystal data	
Chemical formula	[Cd_2_Cl_2_(C_10_H_8_N_2_)_4_](C_9_H_5_N_4_O)_2_·0.81H_2_O
*M*_r_	1305.29
Crystal system, space group	Monoclinic, *P*2_1_/*c*
Temperature (K)	293
*a*, *b*, *c* (Å)	12.425 (5), 13.912 (5), 17.382 (5)
β (°)	104.395 (5)
*V* (Å^3^)	2910.3 (18)
*Z*	2
Radiation type	Mo *K*α
μ (mm^−1^)	0.88
Crystal size (mm)	0.56 × 0.22 × 0.19

Data collection	
Diffractometer	Bruker APEXII CCD
Absorption correction	Multi-scan (*SADABS*; Bruker, 2009 ▸)
*T*_min_, *T*_max_	0.805, 0.846
No. of measured, independent and observed [*I* > 2σ(*I*)] reflections	44121, 11429, 8506
*R*_int_	0.020
(sin θ/λ)_max_ (Å^−1^)	0.778

Refinement	
*R*[*F* ^2^ > 2σ(*F* ^2^)], *wR*(*F* ^2^), *S*	0.043, 0.131, 1.04
No. of reflections	11429
No. of parameters	379
No. of restraints	7
H-atom treatment	H-atom parameters constrained
Δρ_max_, Δρ_min_ (e Å^−3^)	1.54, −0.79

Computer programs: *APEX2* and *SAINT* (Bruker, 2009 ▸), *SHELXS97* (Sheldrick, 2008 ▸), *SHELXL2014* (Sheldrick, 2015 ▸) and *PLATON* (Spek, 2009 ▸).

## supplementary crystallographic information

### Crystal data

**Table:**

[Cd_2_Cl_2_(C_10_H_8_N_2_)_4_](C_9_H_5_N_4_O)_2_·0.806H_2_O	*F*(000) = 1312
*M**_r_* = 1305.29	*D*_x_ = 1.490 Mg m^−^^3^
Monoclinic, *P*2_1_/*c*	Mo *K*α radiation, λ = 0.71073 Å
*a* = 12.425 (5) Å	Cell parameters from 13115 reflections
*b* = 13.912 (5) Å	θ = 1.7–35.4°
*c* = 17.382 (5) Å	µ = 0.88 mm^−^^1^
β = 104.395 (5)°	*T* = 293 K
*V* = 2910.3 (18) Å^3^	Prism, colourless
*Z* = 2	0.56 × 0.22 × 0.19 mm

### Data collection

**Table:**

Bruker APEXII CCD diffractometer	8506 reflections with *I* > 2σ(*I*)
Radiation source: fine focus sealed tube	*R*_int_ = 0.020
φ and ω scans	θ_max_ = 33.6°, θ_min_ = 2.7°
Absorption correction: multi-scan (*SADABS*; Bruker, 2009)	*h* = −17→19
*T*_min_ = 0.805, *T*_max_ = 0.846	*k* = −18→21
44121 measured reflections	*l* = −27→26
11429 independent reflections	

### Refinement

**Table:**

Refinement on *F*^2^	7 restraints
Least-squares matrix: full	Hydrogen site location: inferred from neighbouring sites
*R*[*F*^2^ > 2σ(*F*^2^)] = 0.043	H-atom parameters constrained
*wR*(*F*^2^) = 0.131	*w* = 1/[σ^2^(*F*_o_^2^) + (0.0611*P*)^2^ + 2.0973*P*] where *P* = (*F*_o_^2^ + 2*F*_c_^2^)/3
*S* = 1.04	(Δ/σ)_max_ = 0.003
11429 reflections	Δρ_max_ = 1.54 e Å^−^^3^
379 parameters	Δρ_min_ = −0.79 e Å^−^^3^

### Special details

**Table:**

Geometry. All esds (except the esd in the dihedral angle between two l.s. planes) are estimated using the full covariance matrix. The cell esds are taken into account individually in the estimation of esds in distances, angles and torsion angles; correlations between esds in cell parameters are only used when they are defined by crystal symmetry. An approximate (isotropic) treatment of cell esds is used for estimating esds involving l.s. planes.

### Fractional atomic coordinates and isotropic or equivalent isotropic displacement parameters (Å^2^)

**Table:**

	*x*	*y*	*z*	*U*_iso_*/*U*_eq_	Occ. (<1)
Cd1	0.48583 (2)	0.63830 (2)	0.50037 (2)	0.03951 (6)	
Cl1	0.45118 (7)	0.49495 (4)	0.58640 (4)	0.04984 (15)	
N11	0.4782 (2)	0.75528 (15)	0.59703 (11)	0.0475 (5)	
C12	0.5745 (2)	0.77979 (18)	0.64804 (13)	0.0492 (6)	
C13	0.5741 (4)	0.8450 (2)	0.70916 (18)	0.0701 (10)	
H13	0.6406	0.8625	0.7444	0.084*	
C14	0.4754 (4)	0.8833 (2)	0.7174 (2)	0.0754 (11)	
H14	0.4749	0.9259	0.7585	0.090*	
C15	0.3781 (4)	0.8584 (2)	0.6645 (2)	0.0698 (10)	
H15	0.3105	0.8840	0.6685	0.084*	
C16	0.3835 (3)	0.7944 (2)	0.60539 (17)	0.0599 (7)	
H16	0.3177	0.7773	0.5692	0.072*	
N21	0.66628 (18)	0.67905 (16)	0.57084 (13)	0.0499 (5)	
C22	0.6771 (2)	0.73550 (19)	0.63481 (14)	0.0508 (6)	
C23	0.7818 (3)	0.7514 (3)	0.6852 (2)	0.0857 (12)	
H23	0.7896	0.7898	0.7300	0.103*	
C24	0.8734 (3)	0.7099 (4)	0.6682 (3)	0.0975 (14)	
H24	0.9435	0.7204	0.7015	0.117*	
C25	0.8622 (3)	0.6539 (3)	0.6032 (3)	0.0804 (11)	
H25	0.9236	0.6258	0.5907	0.096*	
C26	0.7561 (3)	0.6397 (2)	0.5559 (2)	0.0618 (8)	
H26	0.7474	0.6006	0.5113	0.074*	
N31	0.46705 (18)	0.74567 (14)	0.39413 (11)	0.0433 (4)	
C32	0.3644 (2)	0.76034 (16)	0.34772 (13)	0.0429 (5)	
C33	0.3454 (3)	0.8242 (2)	0.28427 (16)	0.0615 (7)	
H33	0.2737	0.8342	0.2532	0.074*	
C34	0.4343 (4)	0.8727 (2)	0.2680 (2)	0.0743 (11)	
H34	0.4231	0.9153	0.2256	0.089*	
C35	0.5395 (4)	0.8574 (2)	0.3153 (2)	0.0707 (10)	
H35	0.6005	0.8894	0.3055	0.085*	
C36	0.5519 (3)	0.7931 (2)	0.37774 (18)	0.0575 (6)	
H36	0.6229	0.7826	0.4098	0.069*	
N41	0.29665 (17)	0.65857 (15)	0.43755 (12)	0.0440 (4)	
C42	0.2726 (2)	0.70543 (17)	0.36817 (13)	0.0430 (5)	
C43	0.1653 (3)	0.7022 (3)	0.31844 (19)	0.0650 (8)	
H43	0.1496	0.7327	0.2693	0.078*	
C44	0.0835 (3)	0.6536 (3)	0.3426 (2)	0.0775 (11)	
H44	0.0118	0.6511	0.3100	0.093*	
C45	0.1075 (3)	0.6094 (3)	0.4144 (3)	0.0710 (9)	
H45	0.0525	0.5777	0.4324	0.085*	
C46	0.2157 (2)	0.6125 (2)	0.4601 (2)	0.0580 (7)	
H46	0.2327	0.5809	0.5088	0.070*	
C52	−0.0654 (3)	1.0993 (2)	0.5745 (2)	0.0675 (8)	
C53	−0.0125 (3)	1.1611 (3)	0.5323 (2)	0.0673 (8)	
C51	−0.0135 (3)	1.0558 (3)	0.6467 (2)	0.0742 (9)	0.75 (2)
C511	−0.0821 (7)	1.0188 (7)	0.6947 (6)	0.0706 (19)	0.75 (2)
N511	−0.1340 (8)	0.9934 (8)	0.7366 (7)	0.093 (3)	0.75 (2)
C512	0.1033 (4)	1.0422 (18)	0.6761 (8)	0.0773 (16)	0.75 (2)
N512	0.1970 (5)	1.0327 (16)	0.6983 (9)	0.096 (3)	0.75 (2)
C61	−0.0135 (3)	1.0558 (3)	0.6467 (2)	0.0742 (9)	0.25 (2)
C611	−0.062 (2)	1.041 (2)	0.7125 (12)	0.0706 (19)	0.25 (2)
N611	−0.105 (2)	1.025 (2)	0.7621 (13)	0.093 (3)	0.25 (2)
C612	0.1045 (9)	1.046 (6)	0.664 (2)	0.0773 (16)	0.25 (2)
N612	0.1961 (12)	1.026 (5)	0.676 (3)	0.096 (3)	0.25 (2)
C531	−0.0659 (3)	1.1915 (3)	0.4536 (2)	0.0764 (10)	
N531	−0.1062 (3)	1.2178 (4)	0.3913 (2)	0.1098 (14)	
C532	0.0883 (3)	1.2078 (3)	0.5690 (2)	0.0710 (8)	
N532	0.1683 (3)	1.2487 (3)	0.5991 (2)	0.0916 (10)	
O521	−0.1747 (2)	1.08810 (18)	0.54269 (17)	0.0796 (7)	
C521	−0.2240 (4)	0.9924 (3)	0.5284 (4)	0.0941 (14)	
H52A	−0.2230	0.9709	0.4754	0.113*	
H52B	−0.1811	0.9472	0.5664	0.113*	
C522	−0.3369 (4)	0.9956 (3)	0.5360 (4)	0.1013 (16)	
H52C	−0.3801	1.0371	0.4959	0.152*	
H52D	−0.3377	1.0198	0.5876	0.152*	
H52E	−0.3679	0.9321	0.5297	0.152*	
O71	0.1575 (6)	0.4775 (5)	0.6000 (4)	0.080*	0.403 (6)

### Atomic displacement parameters (Å^2^)

**Table:**

	*U*^11^	*U*^22^	*U*^33^	*U*^12^	*U*^13^	*U*^23^
Cd1	0.04263 (10)	0.03855 (9)	0.03202 (8)	0.00263 (6)	−0.00077 (6)	−0.00296 (5)
Cl1	0.0755 (4)	0.0419 (3)	0.0324 (2)	0.0095 (3)	0.0140 (3)	0.00108 (18)
N11	0.0640 (13)	0.0446 (10)	0.0315 (8)	−0.0059 (9)	0.0077 (8)	−0.0059 (7)
C12	0.0719 (16)	0.0405 (11)	0.0315 (10)	−0.0138 (11)	0.0061 (10)	−0.0023 (8)
C13	0.107 (3)	0.0564 (16)	0.0434 (14)	−0.0262 (17)	0.0129 (16)	−0.0149 (12)
C14	0.131 (4)	0.0483 (15)	0.0536 (17)	−0.0131 (19)	0.036 (2)	−0.0152 (13)
C15	0.109 (3)	0.0493 (15)	0.0586 (18)	0.0133 (16)	0.0361 (19)	−0.0042 (12)
C16	0.0737 (19)	0.0552 (15)	0.0513 (14)	0.0131 (14)	0.0165 (13)	−0.0078 (12)
N21	0.0456 (11)	0.0494 (11)	0.0476 (11)	−0.0068 (9)	−0.0017 (8)	−0.0015 (9)
C22	0.0561 (14)	0.0506 (13)	0.0377 (11)	−0.0178 (11)	−0.0036 (10)	0.0038 (9)
C23	0.073 (2)	0.103 (3)	0.064 (2)	−0.027 (2)	−0.0153 (17)	−0.0182 (19)
C24	0.0536 (19)	0.118 (3)	0.100 (3)	−0.019 (2)	−0.0207 (19)	−0.014 (3)
C25	0.0421 (15)	0.090 (3)	0.100 (3)	−0.0078 (15)	−0.0002 (17)	0.003 (2)
C26	0.0429 (14)	0.0633 (18)	0.074 (2)	−0.0022 (12)	0.0044 (13)	−0.0077 (14)
N31	0.0502 (11)	0.0402 (9)	0.0388 (9)	0.0033 (8)	0.0095 (8)	0.0002 (7)
C32	0.0561 (13)	0.0382 (10)	0.0325 (9)	0.0085 (9)	0.0073 (9)	−0.0023 (8)
C33	0.087 (2)	0.0510 (14)	0.0423 (13)	0.0154 (14)	0.0079 (13)	0.0107 (11)
C34	0.122 (3)	0.0496 (16)	0.0569 (18)	0.0046 (17)	0.032 (2)	0.0134 (13)
C35	0.098 (3)	0.0522 (16)	0.074 (2)	−0.0134 (16)	0.044 (2)	−0.0017 (14)
C36	0.0599 (16)	0.0516 (14)	0.0630 (17)	−0.0032 (12)	0.0191 (13)	−0.0037 (12)
N41	0.0390 (9)	0.0473 (10)	0.0422 (10)	0.0034 (8)	0.0035 (8)	0.0000 (8)
C42	0.0447 (11)	0.0423 (11)	0.0366 (10)	0.0085 (9)	0.0001 (8)	−0.0053 (8)
C43	0.0530 (15)	0.074 (2)	0.0545 (15)	0.0074 (14)	−0.0115 (12)	−0.0021 (14)
C44	0.0452 (16)	0.080 (2)	0.091 (3)	0.0009 (14)	−0.0145 (16)	−0.0062 (19)
C45	0.0438 (14)	0.0609 (17)	0.105 (3)	−0.0066 (13)	0.0133 (16)	−0.0030 (18)
C46	0.0479 (14)	0.0564 (15)	0.0678 (18)	0.0018 (12)	0.0106 (13)	0.0056 (13)
C52	0.0609 (18)	0.0577 (17)	0.087 (2)	0.0086 (14)	0.0240 (16)	−0.0011 (15)
C53	0.0604 (18)	0.0683 (18)	0.076 (2)	−0.0002 (15)	0.0214 (16)	−0.0062 (16)
C51	0.0657 (19)	0.0659 (19)	0.097 (3)	0.0163 (16)	0.0318 (18)	0.0096 (18)
C511	0.066 (4)	0.060 (4)	0.084 (4)	0.009 (3)	0.016 (4)	0.008 (3)
N511	0.086 (4)	0.095 (6)	0.101 (5)	0.015 (3)	0.029 (4)	0.025 (4)
C512	0.073 (2)	0.070 (3)	0.093 (5)	0.0224 (18)	0.028 (2)	0.011 (4)
N512	0.074 (2)	0.099 (4)	0.116 (9)	0.032 (2)	0.026 (3)	0.009 (7)
C61	0.0657 (19)	0.0659 (19)	0.097 (3)	0.0163 (16)	0.0318 (18)	0.0096 (18)
C611	0.066 (4)	0.060 (4)	0.084 (4)	0.009 (3)	0.016 (4)	0.008 (3)
N611	0.086 (4)	0.095 (6)	0.101 (5)	0.015 (3)	0.029 (4)	0.025 (4)
C612	0.073 (2)	0.070 (3)	0.093 (5)	0.0224 (18)	0.028 (2)	0.011 (4)
N612	0.074 (2)	0.099 (4)	0.116 (9)	0.032 (2)	0.026 (3)	0.009 (7)
C531	0.065 (2)	0.090 (3)	0.074 (2)	−0.0169 (18)	0.0172 (17)	−0.0067 (19)
N531	0.094 (3)	0.154 (4)	0.072 (2)	−0.035 (3)	0.0030 (19)	0.009 (2)
C532	0.0626 (19)	0.076 (2)	0.076 (2)	0.0026 (16)	0.0196 (16)	0.0064 (17)
N532	0.0658 (19)	0.107 (3)	0.095 (2)	−0.0156 (18)	0.0062 (17)	0.007 (2)
O521	0.0581 (13)	0.0659 (14)	0.113 (2)	−0.0043 (11)	0.0186 (13)	0.0077 (13)
C521	0.098 (3)	0.073 (3)	0.114 (4)	−0.011 (2)	0.031 (3)	−0.012 (2)
C522	0.093 (3)	0.089 (3)	0.122 (4)	−0.025 (2)	0.027 (3)	0.009 (2)

### Geometric parameters (Å, º)

**Table:**

Cd1—N31	2.341 (2)	C34—H34	0.9300
Cd1—N21	2.342 (2)	C35—C36	1.385 (5)
Cd1—N41	2.350 (2)	C35—H35	0.9300
Cd1—N11	2.358 (2)	C36—H36	0.9300
Cd1—Cl1	2.5920 (9)	N41—C46	1.332 (4)
Cd1—Cl1^i^	2.6289 (8)	N41—C42	1.338 (3)
Cl1—Cd1^i^	2.6289 (8)	C42—C43	1.397 (3)
N11—C16	1.337 (4)	C43—C44	1.371 (5)
N11—C12	1.344 (3)	C43—H43	0.9300
C12—C13	1.398 (4)	C44—C45	1.356 (6)
C12—C22	1.485 (4)	C44—H44	0.9300
C13—C14	1.376 (6)	C45—C46	1.382 (4)
C13—H13	0.9300	C45—H45	0.9300
C14—C15	1.369 (6)	C46—H46	0.9300
C14—H14	0.9300	C52—O521	1.343 (4)
C15—C16	1.375 (4)	C52—C53	1.397 (5)
C15—H15	0.9300	C52—C51	1.398 (5)
C16—H16	0.9300	C53—C532	1.414 (5)
N21—C26	1.326 (4)	C53—C531	1.428 (6)
N21—C22	1.340 (3)	C51—C512	1.425 (6)
C22—C23	1.393 (4)	C51—C511	1.428 (6)
C23—C24	1.372 (6)	C511—N511	1.143 (6)
C23—H23	0.9300	C512—N512	1.139 (6)
C24—C25	1.351 (6)	C611—N611	1.145 (7)
C24—H24	0.9300	C612—N612	1.140 (8)
C25—C26	1.383 (4)	C531—N531	1.135 (5)
C25—H25	0.9300	C532—N532	1.152 (5)
C26—H26	0.9300	O521—C521	1.461 (4)
N31—C36	1.333 (4)	C521—C522	1.442 (7)
N31—C32	1.344 (3)	C521—H52A	0.9700
C32—C33	1.390 (3)	C521—H52B	0.9700
C32—C42	1.488 (4)	C522—H52C	0.9600
C33—C34	1.382 (6)	C522—H52D	0.9600
C33—H33	0.9300	C522—H52E	0.9600
C34—C35	1.377 (6)		
			
N31—Cd1—N21	98.79 (8)	C33—C32—C42	121.9 (2)
N31—Cd1—N41	70.42 (8)	C34—C33—C32	119.1 (3)
N21—Cd1—N41	158.62 (8)	C34—C33—H33	120.4
N31—Cd1—N11	96.20 (8)	C32—C33—H33	120.4
N21—Cd1—N11	70.30 (8)	C35—C34—C33	119.5 (3)
N41—Cd1—N11	92.04 (8)	C35—C34—H34	120.3
N31—Cd1—Cl1	161.37 (6)	C33—C34—H34	120.3
N21—Cd1—Cl1	99.17 (6)	C34—C35—C36	118.1 (3)
N41—Cd1—Cl1	94.01 (6)	C34—C35—H35	120.9
N11—Cd1—Cl1	94.48 (6)	C36—C35—H35	120.9
N31—Cd1—Cl1^i^	89.01 (5)	N31—C36—C35	123.2 (3)
N21—Cd1—Cl1^i^	95.04 (6)	N31—C36—H36	118.4
N41—Cd1—Cl1^i^	102.95 (6)	C35—C36—H36	118.4
N11—Cd1—Cl1^i^	165.01 (6)	C46—N41—C42	119.0 (2)
Cl1—Cd1—Cl1^i^	84.51 (3)	C46—N41—Cd1	123.16 (18)
Cd1—Cl1—Cd1^i^	95.49 (3)	C42—N41—Cd1	116.81 (17)
C16—N11—C12	119.2 (2)	N41—C42—C43	120.5 (3)
C16—N11—Cd1	123.40 (18)	N41—C42—C32	116.9 (2)
C12—N11—Cd1	117.30 (18)	C43—C42—C32	122.6 (2)
N11—C12—C13	119.7 (3)	C44—C43—C42	119.5 (3)
N11—C12—C22	116.8 (2)	C44—C43—H43	120.2
C13—C12—C22	123.6 (3)	C42—C43—H43	120.2
C14—C13—C12	120.1 (3)	C45—C44—C43	119.7 (3)
C14—C13—H13	119.9	C45—C44—H44	120.2
C12—C13—H13	119.9	C43—C44—H44	120.2
C15—C14—C13	119.6 (3)	C44—C45—C46	118.4 (3)
C15—C14—H14	120.2	C44—C45—H45	120.8
C13—C14—H14	120.2	C46—C45—H45	120.8
C14—C15—C16	117.8 (4)	N41—C46—C45	122.9 (3)
C14—C15—H15	121.1	N41—C46—H46	118.5
C16—C15—H15	121.1	C45—C46—H46	118.5
N11—C16—C15	123.6 (3)	O521—C52—C53	114.5 (3)
N11—C16—H16	118.2	O521—C52—C51	120.9 (3)
C15—C16—H16	118.2	C53—C52—C51	124.5 (3)
C26—N21—C22	119.3 (2)	C52—C53—C532	121.6 (4)
C26—N21—Cd1	122.74 (19)	C52—C53—C531	121.2 (3)
C22—N21—Cd1	117.49 (18)	C532—C53—C531	116.5 (3)
N21—C22—C23	120.0 (3)	C52—C51—C512	125.5 (6)
N21—C22—C12	117.3 (2)	C52—C51—C511	118.1 (5)
C23—C22—C12	122.7 (3)	C512—C51—C511	116.4 (5)
C24—C23—C22	119.5 (3)	N511—C511—C51	175.7 (7)
C24—C23—H23	120.2	N512—C512—C51	178.4 (15)
C22—C23—H23	120.2	N531—C531—C53	177.9 (4)
C25—C24—C23	120.2 (3)	N532—C532—C53	177.7 (4)
C25—C24—H24	119.9	C52—O521—C521	120.9 (3)
C23—C24—H24	119.9	C522—C521—O521	109.4 (4)
C24—C25—C26	117.7 (4)	C522—C521—H52A	109.8
C24—C25—H25	121.1	O521—C521—H52A	109.8
C26—C25—H25	121.1	C522—C521—H52B	109.8
N21—C26—C25	123.3 (3)	O521—C521—H52B	109.8
N21—C26—H26	118.4	H52A—C521—H52B	108.3
C25—C26—H26	118.4	C521—C522—H52C	109.5
C36—N31—C32	118.6 (2)	C521—C522—H52D	109.5
C36—N31—Cd1	123.74 (19)	H52C—C522—H52D	109.5
C32—N31—Cd1	117.64 (16)	C521—C522—H52E	109.5
N31—C32—C33	121.5 (3)	H52C—C522—H52E	109.5
N31—C32—C42	116.6 (2)	H52D—C522—H52E	109.5
			
C16—N11—C12—C13	0.5 (4)	C32—C33—C34—C35	−0.5 (5)
Cd1—N11—C12—C13	−176.6 (2)	C33—C34—C35—C36	0.2 (5)
C16—N11—C12—C22	−178.8 (2)	C32—N31—C36—C35	0.3 (4)
Cd1—N11—C12—C22	4.1 (3)	Cd1—N31—C36—C35	−179.1 (2)
N11—C12—C13—C14	0.5 (4)	C34—C35—C36—N31	0.0 (5)
C22—C12—C13—C14	179.7 (3)	C46—N41—C42—C43	−3.1 (4)
C12—C13—C14—C15	−1.1 (5)	Cd1—N41—C42—C43	165.6 (2)
C13—C14—C15—C16	0.8 (5)	C46—N41—C42—C32	176.7 (2)
C12—N11—C16—C15	−0.8 (4)	Cd1—N41—C42—C32	−14.7 (3)
Cd1—N11—C16—C15	176.1 (2)	N31—C32—C42—N41	10.4 (3)
C14—C15—C16—N11	0.1 (5)	C33—C32—C42—N41	−169.1 (2)
C26—N21—C22—C23	−0.7 (4)	N31—C32—C42—C43	−169.9 (2)
Cd1—N21—C22—C23	171.2 (2)	C33—C32—C42—C43	10.6 (4)
C26—N21—C22—C12	178.6 (3)	N41—C42—C43—C44	2.6 (4)
Cd1—N21—C22—C12	−9.5 (3)	C32—C42—C43—C44	−177.1 (3)
N11—C12—C22—N21	3.6 (3)	C42—C43—C44—C45	0.0 (5)
C13—C12—C22—N21	−175.7 (3)	C43—C44—C45—C46	−1.9 (6)
N11—C12—C22—C23	−177.2 (3)	C42—N41—C46—C45	1.1 (4)
C13—C12—C22—C23	3.6 (4)	Cd1—N41—C46—C45	−166.8 (3)
N21—C22—C23—C24	0.8 (6)	C44—C45—C46—N41	1.4 (5)
C12—C22—C23—C24	−178.4 (4)	O521—C52—C53—C532	157.0 (3)
C22—C23—C24—C25	−0.2 (7)	C51—C52—C53—C532	−19.1 (6)
C23—C24—C25—C26	−0.6 (7)	O521—C52—C53—C531	−13.2 (5)
C22—N21—C26—C25	−0.1 (5)	C51—C52—C53—C531	170.6 (4)
Cd1—N21—C26—C25	−171.6 (3)	O521—C52—C51—C512	163.4 (13)
C24—C25—C26—N21	0.7 (6)	C53—C52—C51—C512	−20.7 (14)
C36—N31—C32—C33	−0.7 (3)	O521—C52—C51—C511	−14.6 (7)
Cd1—N31—C32—C33	178.77 (19)	C53—C52—C51—C511	161.4 (6)
C36—N31—C32—C42	179.7 (2)	C53—C52—O521—C521	128.9 (4)
Cd1—N31—C32—C42	−0.8 (3)	C51—C52—O521—C521	−54.7 (5)
N31—C32—C33—C34	0.9 (4)	C52—O521—C521—C522	149.3 (4)
C42—C32—C33—C34	−179.7 (3)		

Symmetry code: (i) −*x*+1, −*y*+1, −*z*+1.

### Hydrogen-bond geometry (Å, º)

**Table:**

*D*—H···*A*	*D*—H	H···*A*	*D*···*A*	*D*—H···*A*
C14—H14···Cl1^ii^	0.93	2.79	3.651 (4)	154
C15—H15···N512	0.93	2.63	3.456 (17)	149
C15—H15···N612	0.93	2.46	3.29 (5)	149
C34—H34···Cl1^iii^	0.93	2.82	3.705 (4)	160
C46—H46···O71	0.93	2.49	3.292 (8)	145

Symmetry codes: (ii) −*x*+1, *y*+1/2, −*z*+3/2; (iii) *x*, −*y*+3/2, *z*−1/2.
